# Cell Cycle Reactivation, at the Start of Neurodegeneration, Induced by Forskolin and Aniline in Differentiated Neuroblastoma Cells

**DOI:** 10.3390/ijms241814373

**Published:** 2023-09-21

**Authors:** Valentina Sturiale, Francesca Bruno, Desiree Brancato, Agata Grazia D’Amico, Grazia Maugeri, Velia D’Agata, Salvatore Saccone, Concetta Federico

**Affiliations:** 1 Department of Biological, Geological and Environmental Sciences, University of Catania, 95124 Catania, Italy; valentina.sturiale@unict.it (V.S.); francesca.bruno@unict.it (F.B.);; 2 Department of Drug and Health Sciences, University of Catania, 95125 Catania, Italy; 3 Department of Biomedical and Biotechnological Sciences, University of Catania, 95123 Catania, Italy

**Keywords:** cell cycle, cyclins, nuclear tau, nucleolus, neurodegeneration, SH-SY5Y cell line, Alzheimer’s disease

## Abstract

A characteristic hallmark of Alzheimer’s disease (AD) is the intracellular accumulation of hyperphosphorylated tau protein, a phenomenon that appears to have associations with oxidative stress, double-stranded DNA breakage, and the de-condensation of heterochromatin. Re-entry into the cell division cycle appears to be involved in the onset of this neurodegenerative process. Indeed, the cell cycle cannot proceed regularly in the differentiated neurons leading to cell death. Here, we induced cell cycle reactivation in neuronal-like cells, obtained by neuroblastoma cells treated with retinoic acid, by exposure to forskolin or aniline. These compounds determine tau hyperphosphorylation or oxidative stress, respectively, resulting in the appearance of features resembling the start of neuronal degeneration typical of AD, such as tau hyperphosphorylation and re-entry into the cell cycle. Indeed, we detected an increased transcriptional level of cyclins and the appearance of a high number of mitotic cells. We also observed a delay in the initiation of the cell cycle when forskolin was co-administered with pituitary adenylate cyclase-activating polypeptide (PACAP). This delay was not observed when PACAP was co-administered with aniline. Our data demonstrate the relevance of tau hyperphosphorylation in initiating an ectopic cell cycle in differentiated neuronal cells, a condition that can lead to neurodegeneration. Moreover, we highlight the utility of neuroblastoma cell lines as an in vitro cellular model to test the possible neuroprotective effects of natural molecules.

## 1. Introduction

Cell cycle progression is a key event in cellular homeostasis. It consists of the G1, S, G2, and M stages whose correct alternation is regulated, at specific checkpoints, by the binding of cyclins and cyclin-dependent kinases (CDKs) to form stable protein complexes that are specifically expressed during the cell cycle, and each of the cyclins can be associated with a different stage [1,2]. For the progression of a cell cycle, which ends in cell division, the proliferating cells must pass the restriction point (R) in the G_1_ phase, which is necessary to start the S phase. The R point is controlled by the binding of cyclin-D to CDK4 and CDK6, and later by cyclin-E to CDK2. Once cells progress beyond the R point, the next S phase begins with cyclin-A/CDK2 complex formation [3]. The G2 phase and its transition to the M phase appear to be triggered by cyclin-A/CDK1 and cyclin-B/CDK1 complexes, as demonstrated by the presence of two peaks of cyclin-A, one peak in the S-phase and the other in G2, and an increase in the levels of cyclin-B during the G2 phase [4]. Thousands of proteins are phosphorylated by CDK1 during the earliest M phase and this kinase is in turn regulated by the cyclin-A and -B families. These events include separation of the centrosomes, chromosome condensation, nuclear envelope disassembly, and mitotic spindle formation. However, the exact mechanisms of cyclin-A and -B actions remain unclear [5]. During differentiation, cells exit from the replicative cell cycle and move to the so-called G0 phase, a non-replicative cellular condition that is extended for short or long periods and characterized by specialized metabolic activities, which are generally related to cell differentiation. In some cases, such as the quiescent T cells, differentiated cells can respond to external proliferation-stimulating factors and can re-enter the G1 phase, while in other cases, a deregulation of the G1/G0 transition can give rise to human genetic diseases, such as cancer [6].

In the cell cycle context, neurons deserve particular attention as the neuronal cells are shaped by the alternation of a variable number of replicative cell cycles followed by a final differentiation event that determines the post-mitotic fate of these cells. Previously proposed hypotheses suggest that the neuronal fate is determined in the last G1 phase, when specific cyclins’/CDKs’ activation/inhibition determines the overcoming of the checkpoint or the start of the G0 phase, and thus the initiation of neuronal differentiation [7]. Previous studies describe how neurons stimulated with certain molecules can try to re-enter the cell cycle before their degeneration and death. This attempt to return to a replicative cell cycle can be evaluated by the increased level of specific markers, such as cyclins and CDKs related to the replicative cell cycle [8]. Thus, the abnormally increasing levels of cell cycle markers in postmitotic cells, like neurons, can be used as an indicator of cell disease. Indeed, in normal conditions, neuronal cells remain in their differentiated state. In contrast, in Alzheimer’s disease (AD), Parkinson’s disease (PD), amyotrophic lateral sclerosis (AML), and perhaps other tauopathies, neurons may try to re-enter the replicative cell cycle, initiating an abortive cell cycle that ends with neuronal death [9]. This process, also called the “cell cycle hypothesis,” may involve tau protein, which suspends its physiological turnover, maybe in the absence of a proper autophagic cascade, resulting in the accumulation of protein aggregates and the formation of neurofibrillary tangles (NFTs), which are a typical sign of AD [10]. In addition to this pathogenesis hypothesis of AD, which involves tau hyperphosphorylation, there is also the amyloid cascade hypothesis, which centers around the accumulation and aggregation of beta-amyloid protein in the brain [11,12].

Tau protein is codified by the *MAPT* gene, known to produce six different main isoforms—with the shortest being composed of 344 amino acids—that are detected in fetal neurons [13]. More precisely, the above hypothesis of neuronal cell degeneration may involve the nuclear tau protein [14,15,16,17,18], a protein whose role within the nucleus and the nucleolus has not yet been clearly defined and some phosphorylated isoforms of which [19] appear to be correlated with cellular aging [20,21] and neuronal differentiation [22]. Tau protein is normally involved in stabilizing microtubules, which are structural components within neurons. In AD and certain other neurodegenerative conditions, tau protein becomes hyperphosphorylated, meaning it has more phosphate groups than usual. This hyperphosphorylation leads to the protein forming abnormal clumps or aggregates inside neurons, which is a characteristic feature of these diseases and can disrupt normal cell function [16,17,18,19].

In the present study, we analyzed neuronal differentiation and degeneration using the SH-SY5Y neuroblastoma cell line, a suitable cell model for our purpose, having previously been described as a tool for neurobiological studies [23,24]. These cells can be used both in their replicative state, namely the typical condition of a cell line with an active cell cycle that determines a cell population doubling in about 20–24 h, and in neuronal like conditions, namely their in vitro differentiation via exposure to retinoic acid, mimicking the behavior of human neurons. Cell differentiation and degeneration were monitored by immunodetection of the nuclear tau epitopes Tau-1 and AT8 and by the expression levels of cyclins. The relevance of Tau-1/AT8 epitopes is related to their position in the tau protein, namely in the Proline Rich Domain (PRD) where the nuclear tau protein seems to bind DNA [15,25,26], therefore playing an important role in the regulation of nuclear/nucleolar activity [21]. These neuronal-like cells were then induced to degenerate in two different ways: by adding forskolin to the cell culture, known as a tau hyperphosphorylation inductor in the Ser202/Thr205 region, namely the AT8 site, and determining changes in the expression level of *CCND1* and *CCNB1* cyclins in murine hippocampal neurons [27]; and by adding aniline, a molecule related to ROS (reactive oxygen species) production and oxidative stress in neuronal tissue [28]. Finally, we analyzed the possibility of counteracting the neurodegenerative process which occurs in the differentiated SH-SY5Y cells induced by forskolin or aniline by treating cells with the neuroprotective peptide pituitary adenylate cyclase-activating polypeptide (PACAP) [29], an endogenous molecule which shows protective effects on several tissues, such as in human corneal endothelium [30]. Indeed, exogenously administered PACAP has shown improvements in cognitive performance in an AD animal model and enhanced motor function in a Parkinson’s disease mouse model [29]. Here, we tested if the use of cell lines, although not perfectly comparable to the in vivo systems, could represent a valid alternative to experiments with animals for biomolecular assays on cell neurodegeneration and to test anti-degenerative molecules. Indeed, one of the problems in the study of anti-degenerative molecules is the need to use laboratory animals, and the subsequent preparation of biological samples after sacrificing the animal.

## 2. Results

### 2.1. Neuronal Differentiation of SH-SY5Y Cells, and Detection of Tau1/AT8 Epitopes

**Figure 1:**
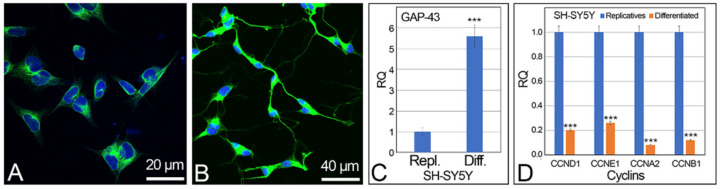
Neuronal differentiation of the SH-SY5Y cells. (**A**) replicative and (**B**) retinoic acid differentiated cells with α-tubulin immunolocalization (green signals). Cell nuclei were stained with DAPI (blue). (**C**) Expression level of the neuronal differentiation marker GAP-43 by qRT-PCR. (**D**) Expression level of cyclins *CCND1*, *CCNE1*, *CCNA2*, *CCNB1* in SH-SY5Y replicative and differentiated cells by qRT-PCR. Scale bars: (**A**) 20 μm; (**B**) 40 μm. RQ: relative quantitation. *** statistical significance, obtained by two tails *t*-test: *p* < 0.001.

SH-SY5Y cells were differentiated in neuronal-like cells by treatment with retinoic acid. This treatment determines the block of the cell cycle, the cell synchronization in the G1 phase, and the start of the neuronal differentiation that was highlighted by modification of the cell morphology and by the presence/absence of replicative vs. differentiated cell markers. Neuronal cell morphology was detected, during the cell culture differentiation process, directly in the culture flasks by using an inverted microscope. After cell processing, cell differentiation was verified by using the α-tubulin immunolocalization, which specifically marks the microtubules in the cytoskeleton, highlighting the neurites of differentiated cells (Figure 1A,B), and by quantitative reverse transcription PCR (qRT-PCR) of the differentiation neuronal marker GAP-43, which shows the increased expression levels in differentiated vs. replicative cells (Figure 1C). The expression levels of the cyclins *CCND1*, *CCNE1*, *CCNA2*, and *CCNB1*, related to the replicative status of the cells, were also detected (Figure 1D), confirming the differentiated status of the retinoic-induced SH-SY5Y cells.

**Figure 2:**
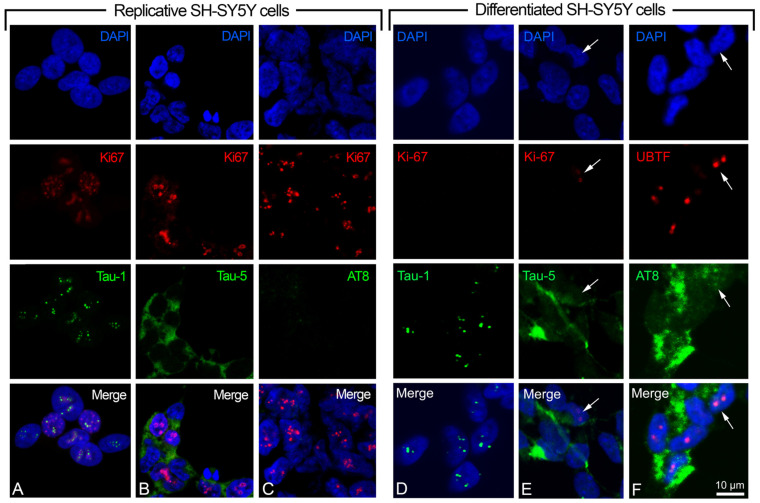
Dual color immunolocalization of tau epitopes in SH-SY5Y cells. (**A**–**C**) Replicative SH-SY5Y cells with the visualization of Tau-1, Tau-5, and AT8 epitopes, respectively. (**D**–**F**) Differentiated SH-SY5Y cells with the visualization of Tau-1, Tau-5, and AT8 epitopes, respectively. Tau-1, Tau-5, and AT8 were revealed by FITC-conjugated antibodies (green signals). Ki-67 (replication marker) and UBTF (nucleolar marker) were detected by TRITC-conjugated antibodies (red signals). DAPI (blue signals) was used to stain cell nuclei. White arrow in (**E**) indicates a cell nucleus with the presence of the Ki-67 marker. White arrow in (**F**) indicates the co-localization of AT8 and UBTF in a nucleus. Magnification is the same for all the images, with a unique scale bar shown in (**F**): 10 μm. The images were captured by confocal laser scanning microscope at 630× magnification. Software to analyze signal co-localization was ZEN-2010 (see Section 4).

Moreover, we used the anti-Tau-5 antibody (detecting phosphorylated and unphosphorylated total tau) as a tau control, and anti-Ki-67 (specific marker located in the nucleus of replicative cells) as a control of the replicative status of the cells. The indirect immunofluorescence (IIF) detection of the nuclear antigen Ki-67 highlighted the active cell cycle in the replicative SH-SY5Y cells, and its absence in the retinoic acid-induced cells indicated the differentiated status of these cells (Figure 2). In some cases (namely in the cells where Ki-67 is absent), the upstream binding transcription factor (UBTF) (instead of Ki-67) was detected by IIF to highlight the nucleolus of the differentiated cells. Concerning Ki-67, we detected, in the differentiated cell samples, a small percentage of positive cells (1.3 ± 0.29, mean ± S.E.M.), possibly corresponding to those cells that did not react to retinoic acid treatment (Figure 2E shows one of these cases).

We analyzed two alternative epitopes of nuclear tau, Tau-1 (protein without phosphorylation in P189/G207 region) and AT8 (protein phosphorylated in S202/T205 region), identifying the same protein domain with or without phosphorylation. The dual color IIF with Tau-1 or AT8, or Tau-5 and Ki-67 (or UBTF), showed that the Tau-1 epitope is localized in the nucleoli of replicative and differentiated cells, with several small signals (Figure 2A,D), whereas AT8 was absent in the replicative (Figure 2C) but present in the differentiated SH-SY5Y cells. In addition to its widespread cytoplasmic presence, the AT8 epitope was also detected in a high percentage of cell nuclei (26.7 ± 2.57, mean ± S.E.M.), where it colocalized with UBTF (Figure 2F shows one of these cases). Tau-5, as expected, was observed as a diffuse signal in both replicative (Figure 2B) and differentiated cells (Figure 2E).

### 2.2. MAPT Gene Expression, and Isoform Detection

**Figure 3:**
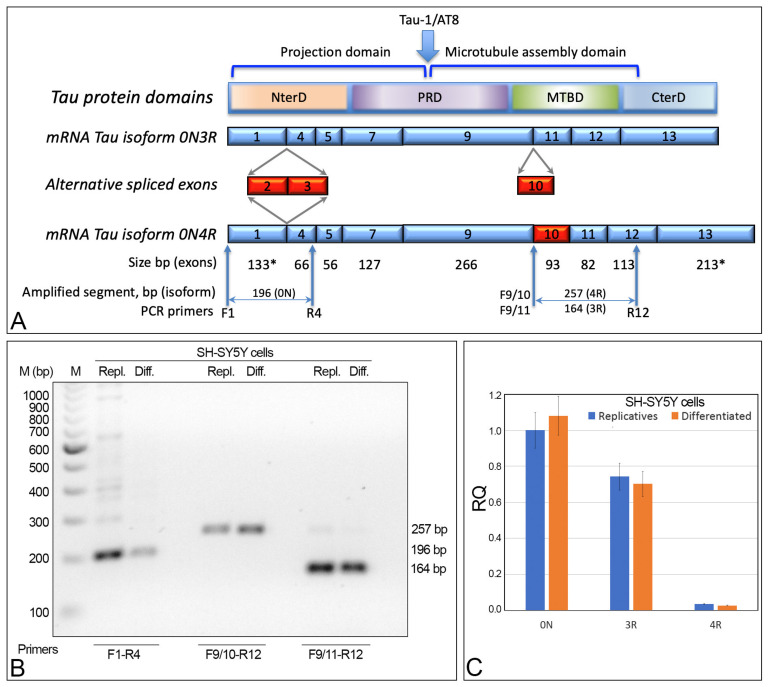
*MAPT* gene structure and alternative splicing analysis. (**A**) Schematic structure of *MAPT* gene with constitutive exons in blue, alternative spliced exons in red. The size (bp) indicated by the asterisk (*) for exon 1 and exon 13 starts with the start codon (exon 1) and ends with the stop codon (exon 13). Exons 4A, 6, and 8 (not detected in SH-SY5Y cells) are not shown. Primers used are indicated by arrows (F: forward and R: reverse). (**B**) Gel electrophoresis showing the DNA segments obtained by RT-PCR amplification of the exons involved in the alternative splicing of the *MAPT* gene, in replicative (Repl.) and differentiated (Diff.) cells. F1-R4 primers detected the 0N tau isoform; F9/10-R12 and F9/11-R12 detected 4R and 3R isoforms, respectively. (**C**) qRT-PCR showing the 0N, 3R, and 4R isoforms in replicative and differentiated cells. RQ: relative quantitation. NS: difference statistically not significant. Primer sequences are shown in Section 4.

To identify the tau isoforms in the replicative and differentiated SH-SY5Y cells, alternative splicing of the *MAPT* gene was analyzed by RT-PCR using specific primers (Figure 3A). F1-R4 primers amplify the sequence between exon 1 and exon 4, and are useful to show the presence/absence of exon 2 and/or 3 (0N, 1N, or 2N isoforms). These primers allowed for the detection, in both replicative and differentiated SH-SY5Y cells, of a DNA segment of 196 bp, matching the 0N isoform. Moreover, 4R (exon 10 present) and 3R (exon 10 absent) isoforms were detected with F9/10-R12 and F9/11-R12 primers, respectively. Both amplifications yielded the expected fragments, indicating that, in replicative or differentiated SH-SY5Y cells, the two mRNAs related to 3R and 4R isoforms are present (those without and with exon 10, respectively) (Figure 3).

Thus, this cell line displays the 0N3R and 0N4R isoforms. The qRT-PCR analysis using mRNA extracted from SH-SY5Y cells confirmed the presence of these two isoforms. More specifically, the 0N, 3R, and 4R isoforms did not show statistically significant differences between the replicative and differentiated cells. Instead, the 3R isoform was detected to be about 25 times more numerous than the 4R isoform. Thus, the most-expressed isoform in replicative and differentiated SH-SY5Y cells is the shortest one (0N3R), with 0N4R having been detected at a very low level (Figure 3C).

Less common tau isoforms were also investigated by looking at the presence of exons 4A, 6, and 8 in the MAPT mRNA. Using specific primers, we did not detect their presence in the replicative or differentiated SH-SY5Y cells.

### 2.3. Effect of Forskolin on the SH-SY5Y Cell Line

Forskolin is a specific protein kinase A (PKA) activator and induces tau hyperphosphorylation in S202/T205 (AT8 epitope), S214, and S396 amino acidic residues [27]. Thus, we analyzed the effect of forskolin in replicative and differentiated SH-SY5Y cells, to test if it alters nuclear tau distribution and/or the expression of cyclins.

**Figure 4:**
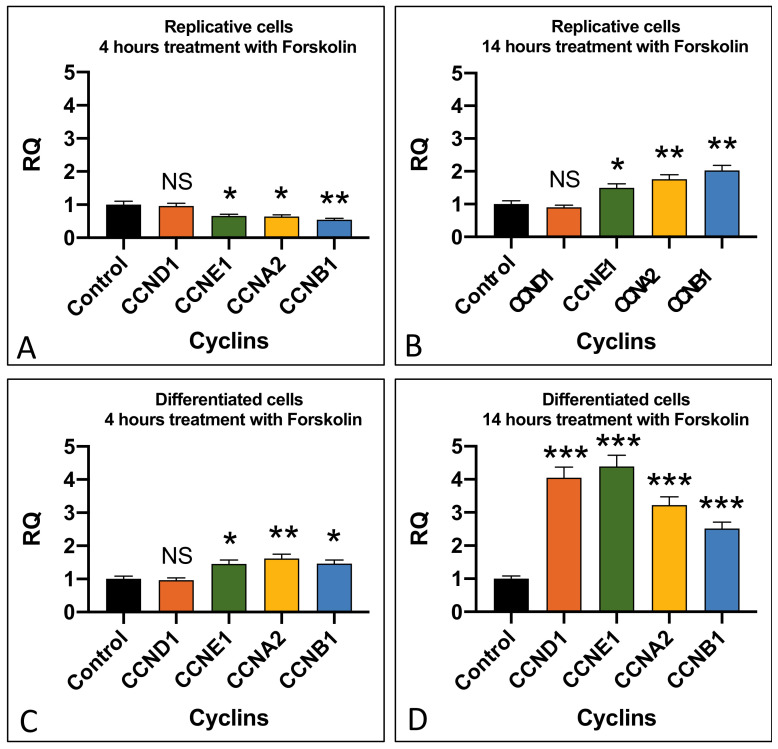
Effect of forskolin in cyclin expression. Cyclin expression in replicative (**A**,**B**) and differentiated (**C**,**D**) SH-SY5Y cells after 4 µM forskolin addition for 4 and 14 h. RQ: relative quantitation obtained by qRT-PCR. Graphs show the results of three independent experiments. Data are expressed as mean ± S.E.M. * *p* < 0.05, ** *p* < 0.01, and *** *p* < 0.001 indicate the statistical significance, obtained by unpaired two-tailed *t*-test, with respect to the control. NS: statistically not significant.

In the replicative cells, we detected a significant increase in the expression of cyclins *CCNE1* (*p* < 0.05), *CCNA2* (*p* < 0.01), and *CCNB1* (*p* < 0.01) after 14 h of cell exposure to forskolin (Figure 4D). In differentiated cells, an increase in the cyclin expression was detected after just 4 h of treatment and it became very high after 14 h of exposure to forskolin, with cyclin *CCND1* (*p* < 0.001) and *CCNE1* (*p* < 0.001) showing the highest levels of expression compared to the untreated cells (Figure 4A–D).

**Figure 5:**
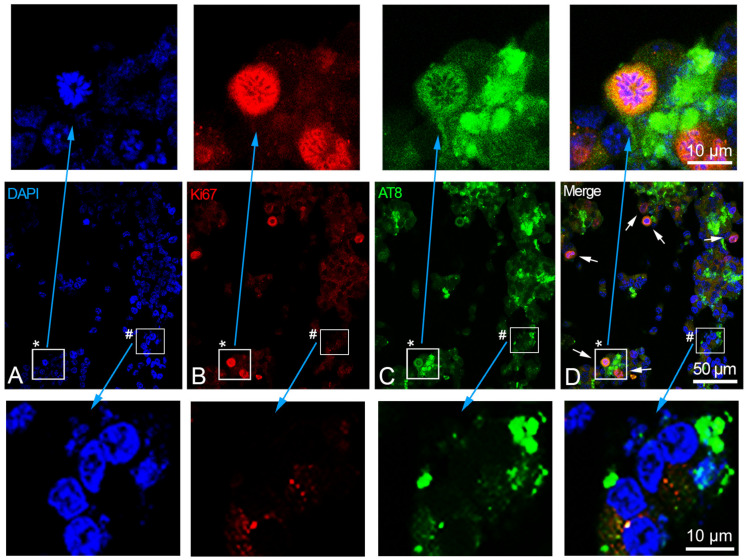
Effect of forskolin in the SH-SY5Y differentiated cells. (**A**–**D**) Immunolocalization of phosphorylated AT8 tau epitope (green signals) and Ki-67 antigen (red signals) in differentiated cells after 14 h of forskolin treatment. Nuclei were stained with DAPI (blue). White arrows in panel (**D**) indicate cells at the metaphase/anaphase stages. (**A**–**C**) The single fluorescence channels of the merged image shown in (**D**). In the upper area of (**A**–**D**), the enlarged sector (the white square indicated by the asterisk) highlights one of the shown cells at the metaphase stage. At the bottom of (**A**–**D**), the enlarged sector (the white square indicated by the symbol #) highlights some cells where AT8 signals are not visible within the nuclei. Images (**A**–**D**) were captured by means of confocal laser scanning microscope at 400× magnification with slice size of 0.5 µm. The upper and bottom enlarged sectors were captured at 630× magnification. Scale bars are indicated in the merge panels.

Nuclear AT8 epitope did not appear altered after forskolin treatment in replicative SH-SY5Y, where AT8 remained undetectable in the cell nuclei before and after exposure to forskolin. Instead, the forskolin treatment (which promotes tau hyperphosphorylation) of differentiated SH-SY5Y cells determined the disappearance of AT8 from the nuclei and its detection only in the cytoplasm (Figure 5). Moreover, we detected the presence of the Ki-67 marker, and the appearance of several mitotic cells (Figure 5), indicating the restart of the cell division cycle in the differentiated SH-SY5Y cells induced by forskolin treatment. More precisely, in the differentiated SH-SY5Ycells after 14 h of forskolin treatment, we detected a percentage of cells with Ki-67 marker (3.7 ± 0.40, mean ± S.E.M.) that was statistically significantly different (*p* < 0.001) from the percentage of Ki-67-positive cells detected in the differentiated cells (1.3 ± 0.29, mean ± S.E.M.). In addition, we detected a percentage of mitotic cells (4.89 ± 0.48, mean ± S.E.M.) that was also statistically significantly different (*p* < 0.001) from the nearly absent mitotic cells (0.22 ± 0.12, mean ± S.E.M.) in the differentiated SH-SY5Y cells.

### 2.4. Effect of Aniline on the SH-SY5Y Cell Line

**Figure 6:**
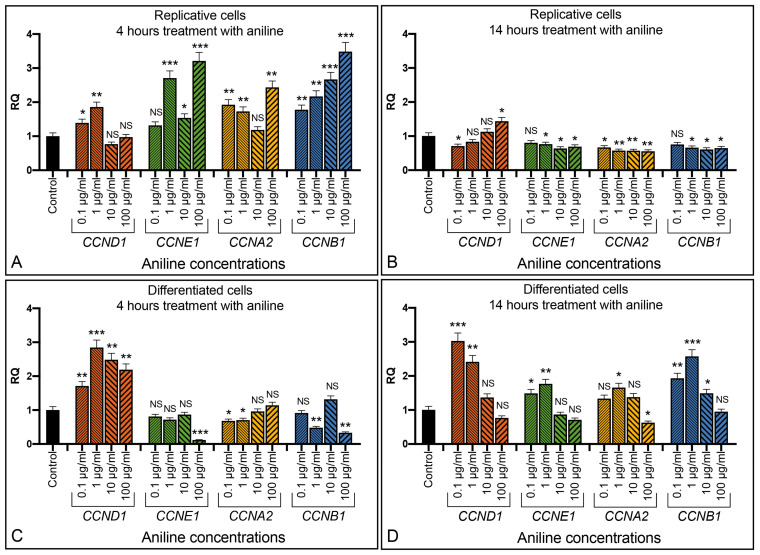
Effect of aniline in the SH-SY5Y cells. Replicative (**A**,**B**) and differentiated (**C**,**D**) cells exposed to different amount of aniline for 4 and 14 h. RQ: relative quantitation evaluated by qRT-PCR. Graphs show the results of three independent experiments. Data are expressed as mean ± S.E.M. * *p* < 0.05, ** *p* < 0.01, and *** *p* < 0.001 indicate the statistical significance, obtained by unpaired two-tailed *t*-test, with respect to the control. NS: statistically not significant.

Aniline is an aromatic amine, widely used in industrial manufacturing, that induces oxidative stress in cells. Replicative SH-SY5Y cells increased expression of cyclins *CCND1*, *CCNE1*, *CCNA2*, and *CCNB1* after few hours of treatment with aniline, and generally in a dose-dependent way (Figure 6A). After 14 h, cyclins returned to levels comparable to the untreated controls (Figure 6B). The aniline treatment of the retinoic acid differentiated SH-SY5Y cells showed, after 4 h, an increased expression level of *CCND1* cyclin (statistical significance *p* < 0.001), with an expression at about twice the level of the initial value being observed (Figure 6C). At 14 h, also, the other cyclins statistically increased the expression level, especially the cyclin *CCNB1* (*p* < 0.001) (Figure 6D).

### 2.5. Blocking of Cell Cycle Reactivation

To understand if the cell cycle re-activation can be reversed or blocked by using bioactive compounds, we tested the effect of PACAP on the differentiated SH-SY5Y cells, where a cell cycle was induced by forskolin or aniline. These treatments were performed for 24 and 48 h, and the expression levels of cyclins *CCND1*, *CCNE1*, *CCNA2*, and *CCNB1* were detected by qRT-PCR.

#### 2.5.1. Cyclins in Control Cells Exposed to Forskolin

**Figure 7:**
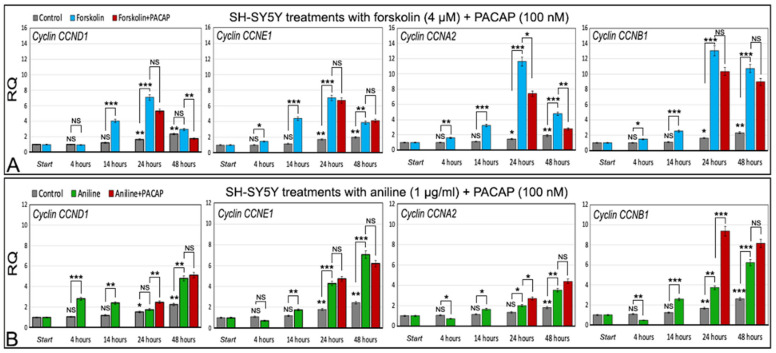
Expression analysis of cyclins in differentiated SH-SY5Y cells treated with forskolin or aniline supplemented with PACAP. (**A**) Forskolin and forskolin/PACAP treatments. (**B**) Aniline and aniline/PACAP treatments. Control: differentiated untreated cells at 0 (start), 4, 14, 24, and 48 h. RQ: relative quantitation obtained by qRT-PCR. Statistical significance was evaluated by two-tail *t*-test. Controls at the various times were compared with respect to the differentiated untreated cells (start). Forskolin or aniline treatments at the various times were compared with the corresponding control at the same treatment time. Forskolin/PACAP or aniline/PACAP treatments at the various times were compared with the corresponding forskolin or aniline treatments, respectively, at the same time. * *p* < 0.05, ** *p* < 0.01, and *** *p* < 0.001. NS: statistically not significant.

In differentiated cells, the expression level of cyclins reaches the highest value after 24 h of exposure to 4 µm forskolin (*p* < 0.001 for all cyclins), and then declines in the next 24 h. The decreasing expression level at 48 h was statistically significant with respect to 24 h for three cyclins: *p* < 0.001 for *CCND1* and *CCNA2*, and *p* < 0.01 for *CCNE1* (Figure 7A, statistical significances between 24 and 48 h are not shown in Figure 7 for ease of reading).

#### 2.5.2. Cyclins in Control Cells Exposed to Aniline

In contrast to forskolin treatment, the expression level of cyclins *CCND1*, *CCNE1*, *CCNA2*, and *CCNB1* induced by 1 µg/mL aniline gradually increased from the start, with the expression level at 48 h always being higher compared to the other times, and with the differences between 24 and 48 h being statistically significant in all cases: *p* < 0.01 for *CCNE1*, *CCNA2*, and *CCNB1*, and *p* < 0.001 for *CCND1* (Figure 7B, statistical significances between 24 and 48 h were not shown in Figure 7 for ease of reading).

#### 2.5.3. Forskolin and PACAP

The four analyzed cyclins, in the cells exposed to forskolin, showed a peak of expression at 24 h, then were decreased at 48 h (Figure 7A). The parallel use of forskolin and PACAP showed, after 24 h, a statistically significant decrease in the expression of cyclin *CCNA2* (*p* < 0.05) with respect to the cells only treated with forskolin. After 48 h, the statistically significant decrease in expression was detected not only for *CCNA2* (*p* < 0.01) but also for cyclin *CCND1* (*p* < 0.01). In the other cases, the differences were not statistically significant (Figure 7A). Thus, at the dose used (100 nM), the PACAP seems to delay the start of cell division cycle reactivation induced by forskolin.

#### 2.5.4. Aniline and PACAP

The four analyzed cyclins, in the cells exposed to aniline, showed a gradual statistically significant increase in expression, reaching the highest value at 48 h (Figure 7B), and the induction of the cell cycle reactivation more slowly with respect to forskolin. The parallel and simultaneous use of aniline and PACAP generally showed low effects on the cyclin expression, with expression differences at 48 h of aniline/PACAP with respect to aniline alone, which was not statistically significant (Figure 7B). Statistically significant differences, between aniline/PACAP vs. aniline alone, were observed at 24 h with cyclin *CCND1* (*p* < 0.01), *CCNA2* (*p* < 0.05), and *CCNB1* (*p* < 0.001), but contrary to the forskolin, here, the cyclin expression was increased (Figure 7B). The PACAP, in this case, seems to synergistically act with aniline in the induction of cyclin expression, at least at 24 h treatment.

## 3. Discussion

In the present study, we used the human neuroblastoma cell line SH-SY5Y to obtain, by means of retinoic acid treatment, neuronal-like differentiated cells in which we induced the re-initiation of the cell cycle, a non-physiological condition that results in cellular degeneration, as previously proposed [8,9]. The restart of the cell cycle was induced by exposing the differentiated SH-SY5Y cells to forskolin or aniline, the first inducing tau hyperphosphorylation, a condition typical of Alzheimer’s disease (AD), and the second inducing oxidative stress in the cells. We then used this system of cell differentiation and degeneration to test the protective effect of a natural compound, the PACAP, on neuronal cell degeneration; more precisely, in contrasting the activation of an ectopic cell division cycle in differentiated cells.

One hypothesis of cell neurodegeneration associated with AD suggests an alteration in the cell cycle with the loss of differentiated status [31,32]. According to this hypothesis, the start of cell degeneration implies that, in differentiated neurons, a cycle of cell division would be activated, which would cause the cells to die as it would not be able to proceed properly through the cell cycle of these postmitotic cells.

The SH-SY5Y and other neuroblastoma cell lines represent a good model for in vitro studies on neuronal differentiation. We previously described, in the differentiated neuroblastoma cell line SK-N-BE, the presence of the AT8 phosphorylated isoform of nuclear tau [22]. Tau is one of the main proteins involved in AD, and the phosphorylated epitopes of the nuclear tau appear to have a central role in aging and at the onset of AD, possibly due to its specific location in the nucleus where it may regulate the transcriptional activity of the nucleolus, as recently described in both human neurons from CA1 region of the hippocampus, and human neuroblastoma SK-N-BE cell line [17,20,22], following several other data on nuclear tau interaction with chromatin [15,16,18,26]. Strictly related to AT8 is the Tau-1 epitope, which corresponds to the same region of AT8 but without phosphorylation and is detected in the nucleolus of both replicative and differentiated cells. The Tau-1 epitope has a different topological organization, possibly related to the different tau protein conformation on the basis of the phosphorylation pattern of the Tau-1/AT8 region [22]. SK-N-BE and SH-SY5Y share the same isoform of nuclear tau as well, namely the smallest one (0N3R), which is largely present in these cells (Figure 3C). The appearance of AT8 epitope in the differentiated SH-SY5Y cell line, together with the disappearance of the active cell cycle marker Ki-67, the increased expression level of the neuronal marker GAP-43, and the decreased expression level of cyclins, was used to highlight the neuronal differentiation induced by retinoic acid (Figure 1 and Figure 2). The expression levels of the cyclins *CCND1*, *CCNE1*, CCNA2, and *CCNB1*, associated with the different checkpoints G0/G1, G1/S, S/G2, and G2/M of the cell cycle, were used to detect cell cycle activation when forskolin or aniline were added to the differentiated SH-SY5Y cells. It should be stressed that, concomitantly to the appearance of cyclins induced by forskolin, AT8 does not disappear with the restart of the cell cycle, but remains in the cytoplasm of the cells (Figure 5). Thus, the restarted cell cycle has important differences from the normal cell division cycle (Figure 2) which portend to the subsequent outcome of cell death.

The start of a cell division cycle induced by the treatment of the differentiated SH-SY5Y cells with forskolin or aniline was monitored by quantifying the cyclin expression. All the cyclin expression data were statistically analyzed with respect to the relative controls, to obtain a clear interpretation of the results (Figure 4, Figure 6 and Figure 7). Thus, we observed a timing of cyclin activation that was coherent with their physiological role in the cells: *CCND1* is the first activated cyclin that is relevant for the checkpoint transition between the G0 and G1 phases, i.e., the transition from the differentiated to the replicative state. After this, the other cyclins, related to the transition between the other cell cycle checkpoints, are activated (Figure 4 and Figure 6). Therefore, using these two molecules, we can induce, in two different ways, a cell cycle in differentiated neuronal cells, which will presumably end with the cell being unable to continue the normal cell cycle and thus with its degeneration.

The described system to induce a cell cycle in neuronal differentiated cells can be useful to test the possible anti-degenerative effects of natural or synthetic molecules in cell cultures. Here, we used PACAP, a neuropeptide with neuroprotective properties, to test this cell model. The co-exposure of the differentiated cells to forskolin and PACAP showed, after 24 and 48 h, that the expression level significantly decreased for cyclin *CCNA2* with respect to the cells exposed only to forskolin, indicating an effect of PACAP in delaying the start of the cell cycle induced by forskolin. In the other cases, the decreased level of expression observed in the cells treated with forskolin/PACAP with respect to the cells treated with forskolin alone was not statistically significant, except in the case of *CCND1* after 48 h of forskolin/PACAP treatment. On the other hand, co-exposure with aniline and PACAP showed different effects, with a general increase in the expression level of cyclins compared to the cells that were only treated with aniline, even if this was statistically significant only at 24 h (cyclins *CCND1*, *CCNA2*, and *CCNB1*); moreover, at 48 h, the differences in the expression of cyclins between cells exposed to aniline/PACAP and to aniline alone were not statistically significant.

Forskolin and aniline showed a general ability to induce a cell division cycle in the retinoic acid differentiated SH-SY5Y cells by means of two different mechanisms. This is also evidenced by the different time of activation of the cyclins, which showed an expression peak at 24 h of treatment with forskolin with respect to a gradual increase up to 48 h of treatment with aniline. Moreover, the use of PACAP further highlighted the two different ways of inducing cell cycle restart, delaying this restart in the case of forskolin and reinforcing it in the case of aniline. Certainly, the molecular events underlying these two different modes of cell cycle induction and the corresponding different actions of PACAP deserve further attention. The PACAP shows different effects in differentiated cells in which a cell cycle was induced based on the hyperphosphorylation of tau (forskolin) or by the induction of oxidative stress (aniline). The results obtained with PACAP are very interesting, as they suggest new prospects in the treatment or prevention of neurodegeneration related to a dysregulation of the cell cycle in differentiated adult neurons.

## 4. Materials and Methods

### 4.1. Cell Cultures, and Neuronal Differentiation

The human neuroblastoma cell line SH-SY5Y, obtained from American Type Culture Collection (ATCC) (Rockville, MD, USA) [33] was grown in DMEM/F12 medium supplemented with 10% heat-inactivated fetal bovine serum (hiFBS) and 1% Penicillin/Streptomycin (100 U/mL; 100 μg/mL) at 37 °C with 5% CO_2_. Neuronal-like cells were obtained from SH-SY5Y cells by 10 μM retinoic acid (RA) treatment (Sigma-Aldrich, Darmstadt, Germany, Cat. N. R2625) and added to the culture medium every 48 h, with gradual serum deprivation (day 0, 1, 3, 5, and 7 with 10%, 2.5%, 1%, 1%, and 0% of serum, respectively). The last RA addition was performed in Neurobasal™ medium (Gibco, Life Technologies, Grand Island, NY, USA, Cat. N. 21103049) with 1% antibiotic Penicillin/Streptomycin, 1X B-27TM Supplement (Gibco, Life Technologies, Grand Island, NY, USA, Cat. N. 17504044) and BDNF (Sigma-Aldrich, Darmstadt, Germany, Cat. N. B3795) [23,24]. Cell differentiation was achieved on the 9th day of treatment. Replicative and differentiated SH-SY5Y were incubated for 4, 14, 24, and 48 h with 4 μM forskolin (Abcam, Cambridge, UK, Cat. N. AB120058) before harvesting, to induce hyperphosphorylation [27,34]. To induce oxidative stress, aniline (Sigma-Aldrich, Darmstadt, Germany, Cat. N. A8524-50G) was added at 0.1, 1, 10 and 100, μg/mL final concentrations for 4 and 14 h as suggested by data from the literature, even if in a different cell type [35]. Moreover, aniline was also used at 1 μg/mL for 24 and 48 h.

To test the effect of PACAP (Sigma-Aldrich, Darmstadt, Germany, Cat. No. A1439), differentiated SH-SY5Y cells were treated with 4 μM forskolin or 1 μg/mL aniline in addition to 100 nM PACAP [36]. The tested time points were 24 and 48 h, 24 h generally being utilized in in vitro cell system studies to evaluate the effects of PACAP [29], to which we further added a longer time (48 h).

### 4.2. Detection of the MAPT Gene Isoforms

RNA from SH-SY5Y was extracted using a MagCore^®^ Compact Automated Nucleic Acid Extractor (RBC Bioscience, New Taipei, Taiwan, Cat. N. MCA0801) and MagCore^®^ Total RNA Cultured Cells Kit (RBC Bioscience, New Taipei, Taiwan, Cat. N. MRC-01). cDNAs were obtained by reverse transcription (SuperScript III First-Strand Synthesis SuperMix, Invitrogen, Thermo Fisher Scientific, Foster City, CA, USA) under the following conditions: 50 °C for 50 min, 85 °C for 5 min. The obtained cDNA was amplified by PCR (Platinum Taq DNA Polymerase, Invitrogen, Thermo Fisher Scientific, Foster City, CA, USA) with the following conditions: 94 °C for 5 min, followed by 35 cycles at 95 °C for 30 s, 60 °C for 30 s, and 72 °C for 40 s. The primers used to identify the exons are shown in Table 1. Amplified DNAs were analyzed by agarose gel electrophoresis, detected by SYBR Safe DNA Gel Stain (Thermo Fisher Scientific, Foster City, CA, USA), and visualized with a Safe imager blue-light transilluminator (Thermo Fisher Scientific, Foster City, CA, USA).

### 4.3. Cyclin Expression Analysis

Detection and quantification of cyclin transcripts were achieved by qRT-PCR with the previously used specific primers (Table 1) using the StepOne instrument (Applied Biosystems, Foster City, CA, USA). Experiments were performed with Power SYBR Green PCR Master Mix (Applied Biosystems, Foster City, CA, USA) according to the manufacturer’s instructions. Experiments were repeated two/three times. The relative quantification method was achieved using actin-b (*ACTB*, Sigma-Aldrich, Darmstadt, Germany) as an endogenous control. Replicative or differentiated SH-SY5Y cells, treated with DMSO (vehicle), were used as calibrator references. Data were analyzed using the Ct value from each sample, normalized with the Ct value from the endogenous control to obtain the ΔCt. The ΔΔCt was obtained by normalizing the ΔCt of the sample of interest with that of the relative calibrator. We then used the 2^−ΔΔCt^ formula to evaluate the relative quantification (RQ) of each sample of interest.

### 4.4. Indirect Immunofluorescence Staining

Indirect immunofluorescence (IIF) analysis was performed on SH-SY5Y cells cultured on glass chamber-slides and fixed in 4% paraformaldehyde for 20 min at room temperature, following the method previously described in [22]. After fixing, cells were washed with phosphate-buffered saline (PBS) and incubated for 15 min in PBS containing 0.5% Triton X-100 (Chemsolute, Hamburg, Germany, Cat. N. 8059.0500). The subsequent step was a preincubation for 1 h at 37 °C with the blocking solution (non-fat dry milk or bovine serum albumin 1%). Then, cells were incubated overnight at 4 °C with the specific primary antibody. Antibodies used were α-Tubulin Polyclonal Antibody (Proteintech Europe, Manchester, UK, Cat. N. 11224-1-AP; 1:100) to detect neuronal processes, Tau-5 (Novus Biologicals, Briarwood Avenue, Centennial, CO, USA, Cat. N. NB200-514; 1:100) to reveal total tau protein, Tau-1 (Millipore, Single Oak Dr, Temecula, CA, USA, Cat. N. MAB3420; 1:100) to detect unphosphorylated tau between Pro189/Gly207 residues, AT8 (Thermo Scientific, Rockford, IL, USA, Cat. N. MN1020; 1:50) to detect pSer202/Thr205 tau, Ki-67 (Invitrogen, Rockford, IL, USA, Cat. N. MA5-14520; 1:100) to discriminate replicative and differentiated cells, and UBTF (Novus Biologicals, Briarwood Avenue, Centennial, CO, USA, Cat. N. NBP1-82545; 1:100) to highlight the nucleolus. After that, cells were additionally washed with PBS and incubated for 1 h at 37 °C with FITC-conjugated anti-mouse secondary antibody (Sigma-Aldrich, Saint Louis, MO, USA, Cat. N. F6257; 1:300) and TRITC-conjugated anti-rabbit (Sigma-Aldrich, Saint Louis, Missouri, USA, Cat. N. T6778; 1:400) to perform the dual color IIF. Experiments were repeated at least three times. The immunodetection was analyzed using a confocal laser scanning microscopy (CLSM) (LSM700, Zeiss, Oberkochen, Baden-Württemberg, Germany) and images were captured at 400× and/or 630× magnification using the ZEN software (ZEN 2010B SP1 v. 6.0.0.485, Zeiss) for image acquisitions and analysis.

Cell counting for statistical analyses was performed as previously described [17,20]. In detail, cells with IIF were counted on 0.5-µm scanned images obtained with CLSM. Three experiments per group were analyzed. The number and percentage of AT8- or Ki67-positive cells and the number of cells at the mitotic stage were obtained in three optical fields, at 400× magnification, for each experiment.

**Figure 8:**
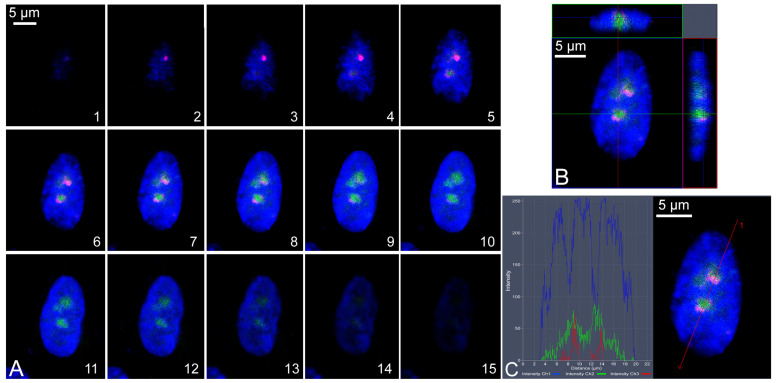
Example of co-localization of IIF signals in the cell nucleus. (**A**) serial section along the z-axis of a cell showing detection of tau (green signals) and ki-67 (red signals). Each image (from 1 to 15) corresponds to a serial section of 0.3 µm thickness. (**B**) Orthogonal view of the same cell shown in (**A**). In the upper part is presented a view of cell section along the green horizontal line. In the right part is presented a view of the cell section along the red vertical line. (**C**) The same cell is shown in (**A**) with the fluorescence intensity (graph on the left) of the tau (green), Ki-67 (red), and DAPI (blue) along the red line indicated by the number 1. The images show the localization in the same nuclear compartment of the analyzed epitopes. Scale bars (5 µm) were indicated for each panel (**A**–**C**). Images were produced with the ZEN-2010 software.

Data regarding co-localization within the same cellular compartment (cytoplasm, nucleus, nucleolus) were obtained using specific functions of the ZEN-2010 software. Indeed, co-localization was achieved through the serial scanning of cells along the z-axis, and the visualization of (1) the scanned serial images, (2) the orthogonal view on the x, y, and z axes, and/or (3) the fluorescence intensity profiles along a defined plane (Figure 8).

### 4.5. Statistical Analysis

The statistical analyses were performed using the software Prism v. 8.0 (GraphPad Software, San Diego, CA, USA, www.graphpad.com (accessed on 27 August 2023)) and Microsoft Excel v. 16.54. The normality of the variables was evaluated by the Kolmogorov–Smirnov test, and statistical differences between pairs of groups were performed by Student’s *t* test. Data are represented as mean ± standard error (S.E.M). The level of significance of the differences between groups was set at: *p* < 0.05 (*), *p* < 0.01 (**), and *p* < 0.001 (***).

## 5. Conclusions

In our work, performed with a neuroblastoma cell line, we have shown how differentiated neuronal-like cells can be easily obtained and induced to re-activate the cell division cycle. This model could be used to test synthetic or natural compounds as well as the efficacy of endogenous neuropeptides, including PACAP, with well-known neuroprotective properties. We think that the in vitro system shown here can be easily replicated in other cell lines to extend studies towards more elaborate models involving, as an example, the relevance of the AT8 epitope of the nuclear tau in neuronal cell differentiation, and its role in neurodegeneration related to the atypical restart of a cell cycle in differentiated neurons. Future work should also verify the presence/absence of this epitope in the brain tissues from AD patients and assess whether it is relevant in in vivo differentiation and in subsequent neurodegeneration of the hippocampal neurons. Nonetheless, despite our results regarding the reactivation of the cell cycle, the potential application of these findings to promising new therapeutic strategies remains limited. This limitation stems from our focus on cell lines, which may not fully replicate the physiology of primary neurons or in vivo brain tissue. Therefore, it would be desirable to continue this work by testing PACAP and other neuroprotective factors on primary cultures and in vivo samples.

## Figures and Tables

**Table 1:** Primers used in this work.

Primers	Type	Nucleotide Sequence 5′-3′	Reference
*MAPT-F1*	Forward	AACCAGGATGGCTGAGCCCC	[37]
*MAPT-R4*	Reverse	GTGACCAGCAGCTTCGTCTT	[37]
*MAPT-F9/10*	Forward	CGGGAAGGTGCAGATAATTAA	[38]
*MAPT-F9/11*	Forward	AGGCGGGAAGGTGCAAATA	[38]
*MAPT-R12*	Reverse	CCCAATCTTCGACTGGACTC	[37]
*CCND1*	Forward	GGATGCTGGAGGTCTGCGA	[39]
	Reverse	AGAGGCCACGAACATGCAAG	
*CCNE1*	Forward	GGTTCCATTTGCCATGGTTA	[40]
	Reverse	CCCTATTTTGTTCAGACAACATGGC	
*CCNA2*	Forward	AGGGAAATGGAGGTTAAATG	[41]
	Reverse	CACTGACATGGAAGACAGGAACCT	
*CCNB1*	Forward	AATGAAATTCAGGTTGTTGCAGGAG	[42]
	Reverse	CATGGCAGTGACACCAACCAG	
*ACTB*	Forward	GACGACATGGAGAAAATCTG	Sigma-Aldrich
	Reverse	ATGATCTGGGTCATCTTCTC	
*GAP-43*	Forward	GAGGAAAAATCTTCAGAGACC	[43]
	Reverse	AACCCTTGAAATCCAGAAAG	

## Data Availability

Not applicable.
